# Referral Patterns to Pediatric Rheumatology From Primary Care Physicians and General Pediatrics at a Single Pediatric Rheumatology Center in Saudi Arabia

**DOI:** 10.7759/cureus.43594

**Published:** 2023-08-16

**Authors:** Sulaiman Alhumaid, Abdulaziz Alsuhibani, Abdulmajeed A Alsabr, Abdulmajed Alrajeh, Yazeed Alhumaidi, Wafaa Al Suwairi

**Affiliations:** 1 College of Medicine, King Saud Bin Abdulaziz University for Health Sciences, Riyadh, SAU; 2 Collage of Medicine, King Saud Bin Abdulaziz University for Health Sciences, Riyadh, SAU; 3 Pediatric Rheumatology, King Abdullah Specialized Children's Hospital (KASCH) National Guard Health Affairs, Riyadh, SAU

**Keywords:** saudi arabia, rheumatic diseases, pediatric rheumatology, juvenile idiopathic arthritis, referral pattern

## Abstract

Objective

This study aims to investigate referral patterns to pediatric rheumatology and assess the correctness of referrals from primary care physicians and pediatric specialties.

Methodology

A cross-sectional, retrospective study was conducted on all patients who were referred to the Pediatric Rheumatology Department since 2015 (*N *= 282) at King Abdullah Specialized Children’s Hospital (KASCH), Pediatric Rheumatology Clinic. Age, gender, reason for referral, clinical features, referring department, and final diagnosis were taken as variables. Data were collected through the documents and records of the cases (referrals) in the electronic medical records system of the hospital (BestCare). Then Excel was used for data entry, and JMP statistical software, version 14.0.0 (SAS Institute Inc., Cary, NC, USA) was used for data analysis.

Results

In a total of 282 patients across the Pediatric Rheumatology Clinic, KASCH, the most common reason for referral to the clinic was joint pain (112, 43%) and the least common reason was rash (6, 2.3%). The most common diagnosis was juvenile idiopathic arthritis (JIA) (24, 26.6%). The majority of patients referred to the rheumatology department did not have a rheumatological disease (169, 65%). The majority of the referrals were from pediatrics subspecialties (168, 65%). The least referred department was primary care ( 21, 8%).

Conclusions

To our knowledge, this is the first study showing the referral pattern, accuracy, and profile of a pediatric rheumatology clinic population in Saudi Arabia. Expectedly, the most common reason for referral was arthralgia. The most common diagnosis was JIA. According to the results, most of the referrals were inaccurate as they did not end up with a rheumatological diagnosis. Pediatric subspecialties should be more aware of the nature of rheumatological disease to avoid over-referrals. Finding a pattern of referrals to pediatric rheumatology is an excellent modality to accomplish early diagnosis and the best possible prognosis.

## Introduction

Pediatric rheumatology is a subspecialty of pediatrics dealing with autoimmune and autoinflammatory diseases affecting vascular tissues, connective tissues, and the musculoskeletal system. Children having complaints due to nonrheumatic conditions are referred to pediatric rheumatology centers for the differential diagnosis of rheumatic diseases [1]. 

Due to the progressive nature of these diseases, any delay or omission in the diagnosis or a delay in the initiation of treatment significantly worsens the prognosis for the patients. Many of the patients face setbacks in their initiation of the treatment or oversights in the diagnosis. It has been reported that the time between symptom onset and diagnosis of rheumatoid arthritis by a rheumatologist in Saudi Arabia can be as high as 30 months [1]. Another example is a Childhood Arthritis Prospective (CAP) study in the United Kingdom, which reported that the time between symptom onset and the first pediatric rheumatology appointment was as high as 58 weeks [2].

While at the onset of their symptoms, patients do not go to see a rheumatologist. They first seek a consultation from primary care physicians. The threshold of referring differs from one primary care physician to another, which disrupts the optimal use of specialists, causing a failure of referrals of patients to rheumatologists soon enough [3]. Dealing with children displaying signs and symptoms of rheumatic diseases and knowing the profile of the patient population in pediatric rheumatology clinics may be helpful for both pediatricians and other referring physicians.

Until now, data from the United Kingdom (national) [4], the United States (national and regional) [5,6], Canada (national and single center) [7-9], Southeast Asian countries (national) [10], and Turkey [11] had been reported. It has been observed that the prevalence of pediatric rheumatic diseases varies geographically.

Successive studies have proven that patients who have been diagnosed lately have poor responses to treatment in comparison to those who had an earlier diagnosis [12]. Delayed specialist referrals constitute a principal reason for late diagnosis and subsequent treatment in Saudi Arabia [13]. So, to reach a well-timed diagnosis, an improvement in the transition from primary care center to a specialist is essential [1]. Finding a pattern of referral to pediatrics rheumatology from primary care physicians or general pediatricians is a great modality to accomplish early diagnosis.

## Materials and methods

A retrospective cross-sectional study was conducted at King Abdullah Specialist Children's Hospital (KASCH), Pediatric Rheumatology Clinic, to investigate the profile of referrals and the accuracy of referrals of all patients who were referred to the clinic between 2015 and 2022. The study subjects included all patients who had been referred to the Pediatric Rheumatology Clinic since the start of the electronic medical records system in 2015 (282 patients) and had no exclusion criteria. The sampling technique utilized for the study was nonprobability consecutive sampling, including all patients who met the inclusion criteria. Data were collected by chart review using the BestCare system at KASCH, and only the research team members collected the data. The collected data included patient demographics, reasons for referral, clinical features, referring departments, and final diagnosis.

The data collected were managed and analyzed using Microsoft Excel and JMP statistical software, version 14.0.0 (SAS Institute Inc., Cary, NC, USA). The categorical data were presented using percentages and frequencies. The chi-square test was used for inferential statistics to find the association between categorical variables. The study adhered to ethical considerations by assuring the privacy and confidentiality of the subjects. An institutional review board (IRB) approval was obtained (SP20/430/R). Informed consent was not required as it was a retrospective study, and no identification data such as medical record numbers, names, and IDs were collected. The access to research data was kept only between the study group members. Overall, this study provided valuable insights into the profile of referrals in the specified study population.

## Results

Demographic characteristics

**Figure 1:**
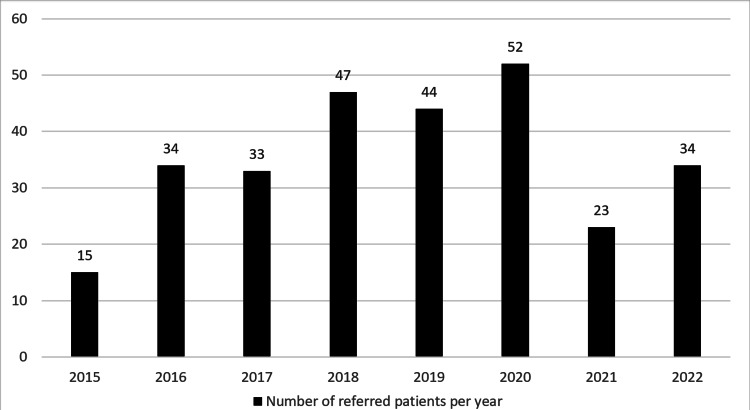
Number of referred patients per year.

A total of 282 new referrals to the Pediatric Rheumatology Clinic were evaluated (Figure 1). The overall gender was nearly equal (146 females and 136 males) (Table 1). The majority of patients referred were adolescents (12+ years; *n *= 131, 47%) (Table 1). A total of 23 patients did not have a follow-up. The average waiting time after referrals of the patients was 13 days.

**Table 1:** The distribution of gender and patients' age among referrals to the pediatric rheumatology outpatient clinic.

Demographic	Count, *n*	%
Male	136	48
Female	146	52
Early childhood (3-8 years)	35	12
Middle childhood (9-11 years)	116	41
Adolescent (12+ years)	131	47

Specialties of referring physicians

A retrospective chart review of 282 referrals was conducted, with 23 patients excluded due to a lack of follow-up. The majority of the referred patients were from pediatric subspecialties (*n *= 168, 65%), and the least referrals were from primary care (*n *= 21, 8%). Pediatric subspecialties had the highest number and percentage of incorrect referrals (*n *= 98, 62%) (Table 2). The most common cause of referrals was joint pain in 43% (*n* = 112) of the patients, followed by referral for evaluation (*n *= 82, 31.6%) (Table 3).

**Table 2:** The distribution of referring physicians to the pediatric rheumatology outpatient clinic according to specialties and whether the patients ended up with a rheumatological disease or not.

Count row	Without a rheumatic disease, *n (*%)	With a rheumatic disease, *n (*%)	Total, *n (*%)
Primary care	13 (58)	8, (42)	21 (8)
General pediatrics	43 (61.4)	27 (38.6)	70 (27)
Pediatric subspecialties	113 (67)	55 (33)	168 (65)
Total	169	90	259

**Table 3:** The distribution of common clinical features for new referrals to the pediatric rheumatology outpatient clinic.

Reason for referral	Total, *n* (%)	Rheumatological disease		*P*-value
		Yes	No	
Joint pain	112 (43)	43	69	0.07
Swelling	24 (9.2)	6	18	0.95
Concurrent care	38 (14.6)	14	24	0.8
Abnormal labs	20 (7.7)	6	14	0.86
Evaluation	82 (31.6)	24	58	0.66
Fever	9 (3.5)	4	5	0.15
Rash	6 (2.3)	3	3	0.56
Movement abnormality	16 (6.2)	7	9	0.08

Final diagnosis of patients

The majority of patients referred to the rheumatology department did not have a rheumatological disease (*n *= 169, 65%). The highest number of incorrect referrals was for joint pain 61% (*n *= 69). The least number of incorrect referrals was for rash (*n *= 3). Patients referred for rash had the highest successful referral percentage (50%). Among patients referred for evaluation, 71% (*n *= 58) did not have a rheumatological disease, which is the highest percentage of wrong referrals. Out of the 90 patients diagnosed with rheumatological disease, the most common diagnosis was juvenile idiopathic arthritis (JIA, *n *= 24, 26.6%). Followed by joint hyperlaxity (*n *= 6, 6.6%). Then, equally, enthesitis-related arthritis (*n *= 4, 4.4%), systemic lupus erythematosus (*n *= 4, 4.4%), immune thrombocytopenia (*n *= 4, 4.4%), rheumatic fever (*n *= 4, 4.4%), Kawasaki disease (*n *= 4, 4.4%), and Behcet’s disease (*n *= 4, 4.4%) (Table 4).

**Table 4:** The final diagnosis of patients with rheumatic disease referred to the pediatric rheumatology outpatient clinic. SLE, systemic lupus erythematosus; ITP, immune thrombocytopenia; GVHD, graft-versus-host disease; CRMO, chronic recurrent multifocal osteomyelitis; PFAPA, periodic fever, aphthous stomatitis, pharyngitis, and adenitis; HLH, hemophagocytic lymphohistiocytosis

Rheumatic diseases	Total (*N* = 90), *n* (%)
Juvenile idiopathic arthritis	24 (26.6)
Joint hyperlaxity	6 (6.6)
Kawasaki disease	4 (4.4)
Enthesitis-related arthritis	4 (4.4)
SLE	4 (4.4)
ITP	4 (4.4)
Behcet's disease	4 (4.4)
Rheumatic fever	4 (4.4)
Sarcoidosis	3 (3.3)
Autoinflammatory syndrome	3 (3.3)
Fibromyalgia	3 (3.3)
GVHD	2 (2.2)
IgA vasculitis	2 (2.2)
Scleroderma	2 (2.2)
Primary Raynaud's phenomenon	2 (2.2)
CRMO	2 (2.2)
Vogt-Koyanagi-Harada disease	2 (2.2)
Complex regional pain syndrome	1 (1.1)
Autoimmune thyroid disease	1 (1.1)
PFAPA syndrome	1 (1.1)
Autoimmune encephalitis	1 (1.1)
Sjogren syndrome	1 (1.1)
Secondary HLH	1 (1.1)
Uveitis	1 (1.1)
Osgood-Schlatter syndrome	1 (1.1)
Reactive arthralgia	1 (1.1)
MTHFR deficiency	1 (1.1)
Impingement syndrome	1 (1.1)
TBK1 gene mutation	1 (1.1)
Skin vasculitis	1 (1.1)
Idiopathic intracranial hypertension	1 (1.1)
Drug-induced lupus	1 (1.1)

Analytical analysis

Rash was the least common pattern seen in referrals (*n *= 6, 2%) (Table 3). Primary care doctors had the highest successful percentage of referrals (*n *= 8, 42%) (Table 2). There were a few patients who did not have a follow-up (*n *= 23), so their data were not analyzed for final diagnosis.

## Discussion

Pediatric rheumatology is a pediatric specialization that focuses on assessing and treating inflammation affecting multiple systems in children, as well as triaging and managing musculoskeletal pain complaints. As more diseases are identified and some autoimmune or inflammatory diseases that affect specific organs require pediatric rheumatologist expertise in co-managing immunosuppressive or biological therapies, this field has grown [14]. Our report is the first to describe the populations of pediatric rheumatology clinics in KASCH for which data were collected. We made numerous novel observations, which we will explore in detail below. This is the first study in Saudi Arabia assessing the profile of the pediatric rheumatology outpatient clinic referrals population. We determined the rate of rheumatic and nonrheumatic diseases of children referred to the unit for the first time since the rheumatology clinic was opened. Among all referrals, only 35% were diagnosed with rheumatic disease and 65% had nonrheumatic conditions.

Referrals from primary care physicians to pediatric rheumatologists only accounted for 11% of all referrals. Referrals from primary care physicians to pediatric rheumatologists are a crucial step in the care of patients with rheumatic diseases. Similarly, previous research papers indicated that only a small fraction of patients are referred from primary care to rheumatology specialists [11]. This lack of referral can lead to delayed diagnosis and treatment, potentially resulting in adverse outcomes for patients. Various factors may contribute to low referral rates, such as a lack of awareness among primary care physicians regarding the clinical features of rheumatological diseases or the availability of rheumatology specialists. Therefore, it is crucial to enhance education and awareness among primary care physicians about the necessity of timely referral and to provide them with appropriate resources to identify and refer patients who require specialist care. By increasing referral rates, patients can receive timely and appropriate care, which may improve their overall quality of life and outcomes [15].

Second, the majority of referrals (146, 52%) were from pediatric subspecialties, similar to what was found in previous studies [11]. However, only 38% of those patients ended up with a confirmed rheumatological diagnosis. Pediatric subspecialists refer a substantial number of patients to rheumatology specialists, although a considerable percentage of these patients do not have a rheumatological disease, which is considered a wrong referral. There could be several reasons for this tendency, including the overlap of musculoskeletal symptoms with nonrheumatological conditions that may be challenging to diagnose for general pediatricians. Another reason could be a lack of specialized knowledge in musculoskeletal health, leading to a higher referral rate of patients with musculoskeletal complaints to rheumatology clinics. Additionally, a cautious approach stemming from the lack of knowledge to avoid missing a possible diagnosis may lead to over-referral of patients, increasing the likelihood of unnecessary testing and interventions. It is essential to increase awareness by looking for educational needs at the level of physicians and recognizing the significance of early diagnosis, accurate referral, and collaboration with other specialists to optimize patient care and avoid unnecessary burdens on healthcare systems [16].

The most frequent reason for referral was joint pain (43%) similar to other studies [1-8], which is expected since joint pain overlaps with various conditions, whether it is rheumatoid, autoimmune, mechanical, infections, or injuries. The least common reason for referral was rash, with only 2% of patients being referred for this issue. This percentage is lower compared to the referrals observed in other studies [11]. JIA was the most common diagnosis encountered with 13 patients. Several studies have reported that JIA is the most common rheumatological disease encountered in pediatric rheumatology clinics, which is consistent with the findings of our paper [2-11]. JIA is a chronic disease that affects children and adolescents, with various subtypes and variable clinical manifestations, making diagnosis and management challenging. Early and accurate diagnosis of JIA is crucial to prevent long-term complications such as joint damage and disability. Therefore, understanding the epidemiology and clinical features of JIA is essential for optimal management and provision of appropriate care for affected children [17]. The consistent findings across multiple studies [2-11] emphasized the significance of ongoing efforts to improve the diagnosis, treatment, and management of JIA. Raising awareness of the disease among healthcare providers, patients, and families is also important to ensure timely referral and early diagnosis.

This study was conducted in a single center. The study's data collection from a solitary center presents limitations to the generalizability of its discoveries. To achieve more accurate and robust results, additional investigations and research are imperative at both local and international levels. Incorporating multiple centers and a larger sample size in future studies would provide a more comprehensive understanding of the subject matter.

## Conclusions

In conclusion, we believe it is important to highlight that this study marks a significant milestone as the first of its kind to explore the referral patterns, diagnostic accuracy, and demographic profile of patients within a pediatric rheumatology clinic setting in Saudi Arabia. Notably, the most common reason for referral was arthralgia, while the predominant diagnosis observed was JIA. However, a noteworthy revelation emerged from the analysis: the majority of the referrals did not end up with a rheumatological diagnosis. This underscores the need for heightened awareness among general pediatricians and family doctors regarding rheumatological conditions, to curtail instances of excessive referrals and shortage of referrals. The identification of referral patterns within pediatric rheumatology offers a promising avenue to facilitate early diagnoses and subsequently optimize prognoses, contributing to the overarching objective of improved patient outcomes. As the findings shed light on this vital aspect of healthcare delivery, it is hoped that further research and collaborative efforts will build upon these insights, ultimately fostering a more informed and effective approach to addressing pediatric rheumatological challenges.
